# Supplementary material for: Threshold-dependent negative autoregulation of PIF4 gene expression optimizes growth and fitness in Arabidopsis
Source: PLoS Genet. 2025 Aug 11;21(8):e1011758. doi: 10.1371/journal.pgen.1011758 (PMC12338842; doi:10.1371/journal.pgen.1011758)
Supplement: S1 Fig — (PDF) [file pgen.1011758.s001.pdf]

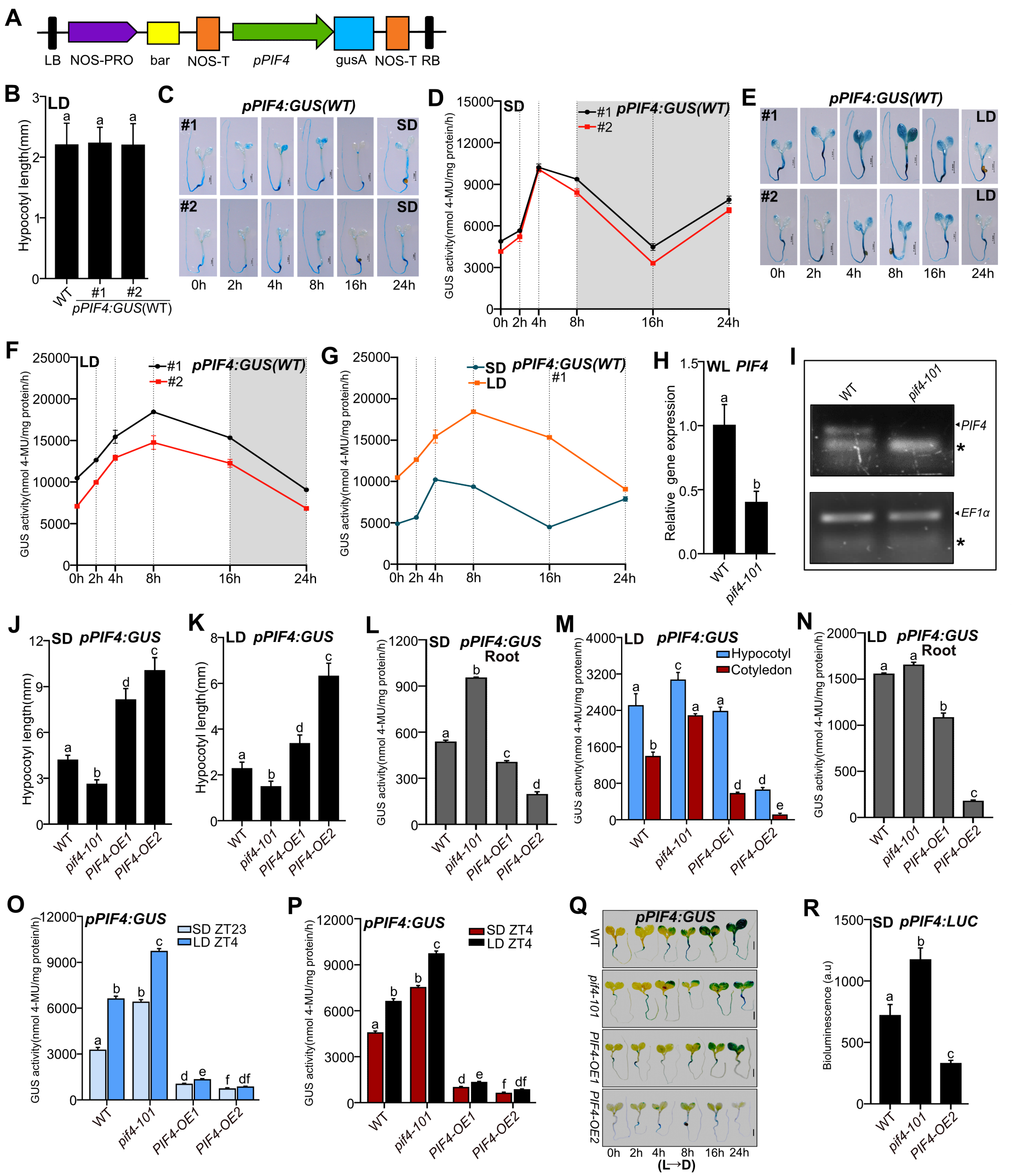

S1 Fig. PIF4 negatively autoregulates its own gene expression in a photoperiod-dependent manner.

(A) Schematic diagram of the construct used for making *pPIF4:GUS*.

(B) Two independent *pPIF4:GUS* lines under the WT background are used. Hypocotyl length of six-day-old WT, *pPIF4:GUS* line #1 and #2 grown in LD under 22°C.

(C-F) GUS stained images and GUS activities from *pPIF4:GUS* line #1 and #2 grown under SD (C-D) and LD (E-F) conditions, respectively at 22°C. Tissue was harvested at ZT0 on the sixth day and grown for 24 hours. The dark period is shown in grey in the graph.

(G) Measurement of GUS activity of *pPIF4:GUS* line #1 grown under SD and LD in diurnal conditions at 22°C.
(H) The PIF4 transcript level was measured by qPCR in six-day-old WT and *pif4-101* mutants grown in SD at ZT23 (22°C). The transcript level was normalized to WT.
(I) Semi-quantitative PCR analysis of PIF4 for WT and *pif4-101* mutant. The diluted cDNA was run in a 2% agarose gel. The asterisk denotes a non-specific band. EF1α was used as a loading control.
(J and K) Hypocotyl length of WT, *pif4-101*, *PIF4-OE1* and *PIF4-OE2* carrying the transgene *pPIF4:GUS* of six-day-old seedlings grown in SD (J) and LD (K) at 22°C.
(L) Root was dissected from ten-day-old whole seedlings of indicated genotypes grown in vertical plates under SD conditions (ZT23) at 22°C, and quantitative GUS activity was measured from the roots.
(M and N) The GUS activity in hypocotyl, cotyledon (M) and root (N) harvested from six-day-old seedlings under LD at 22°C (tissue was harvested at ZT4).
(O) GUS activity was calculated from six-day-old seedlings grown under SD and LD. Tissue was harvested at the end of the night for SD (ZT23) and during the day for LD (ZT4) at 22°C.
(P) Quantification of GUS activity from six-day-old seedlings grown under SD and LD in WL (ZT4) at 22°C.
(Q) GUS-stained images of constant light (22°C) grown seedlings shifted to dark for indicated genotypes at various intervals.
(R) Microplate bioluminescence detection of WT, *pif4-101* and *PIF4-OE2* lines expressing the *pPIF4:LUC*. The six-day-old seedlings were grown in SD photoperiod at 22°C. Seedlings were harvested at the end of the night (ZT23). Values represent mean ± SD, n=3 biological replicates of the 1-s absolute bioluminescence of at least 24 seedlings per genotype. The unit of bioluminescence was represented as an arbitrary unit (a.u.).
Data represent mean±SD; n≥20 for hypocotyl length, n ≥ 40 seedlings for GUS activity data, n=3 biological replicates for gene expression analysis. Different letters indicate a significant difference (one-way and two-way ANOVA with Tukey's HSD test, $P<0.05$). The experiment was repeated three times with similar results. Related to Fig 1.
