# Supplementary material for: Threshold-dependent negative autoregulation of PIF4 gene expression optimizes growth and fitness in Arabidopsis
Source: PLoS Genet. 2025 Aug 11;21(8):e1011758. doi: 10.1371/journal.pgen.1011758 (PMC12338842; doi:10.1371/journal.pgen.1011758)
Supplement: S3 Fig — (PDF) [file pgen.1011758.s003.pdf]

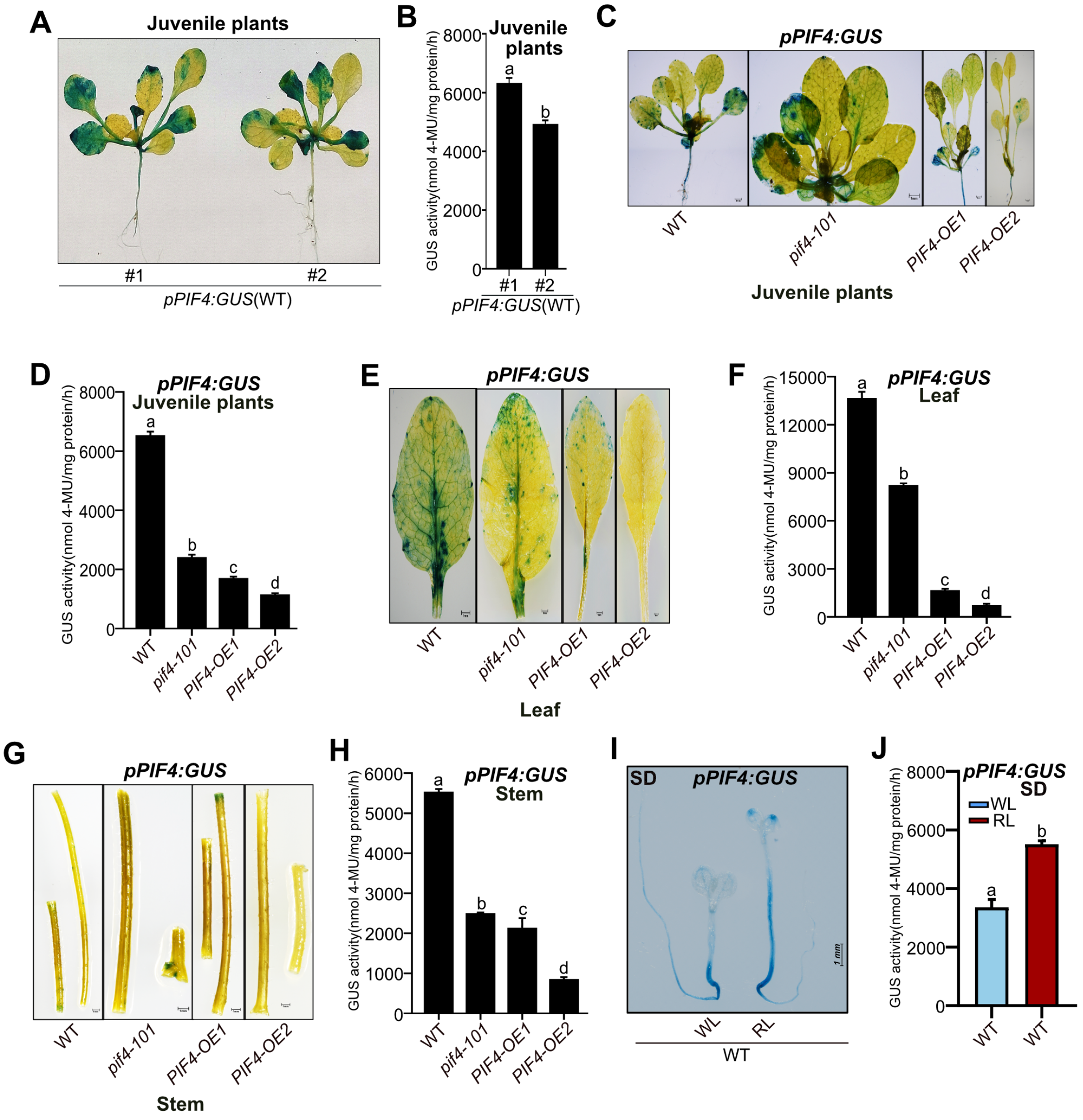

S3 Fig. Autoinhibition of the *PIF4* promoter persists in the adult stage.
(A and B) GUS stained images (A) and GUS activity (B) of three-week-old juvenile plants of two independent *pPIF4:GUS* lines #1 and #2 under WT background grown in 22°C LD conditions.
(C-H) Tissue-specific expression patterns. Representative GUS staining and activity in three-week juvenile plants (C and D) and in rosette leaves (E and F) and stem (G and H) of six-week-old adult plants grown at 22ºC under LD, and tissue was harvested at ZT4. Data represent mean±SD (n=6).
(I and J) GUS staining and activity measurement of six-day-old seedlings of *pPIF4:GUS* grown under WL and RL at SD (22°C). Tissue was harvested at ZT23.
Different letters indicate significant differences (one-way ANOVA with Tukey's HSD test, P < 0.05). The experiment was repeated three times with similar results. Related to Fig 1.
