# Supplementary material for: Threshold-dependent negative autoregulation of PIF4 gene expression optimizes growth and fitness in Arabidopsis
Source: PLoS Genet. 2025 Aug 11;21(8):e1011758. doi: 10.1371/journal.pgen.1011758 (PMC12338842; doi:10.1371/journal.pgen.1011758)
Supplement: S4 Fig — (PDF) [file pgen.1011758.s004.pdf]

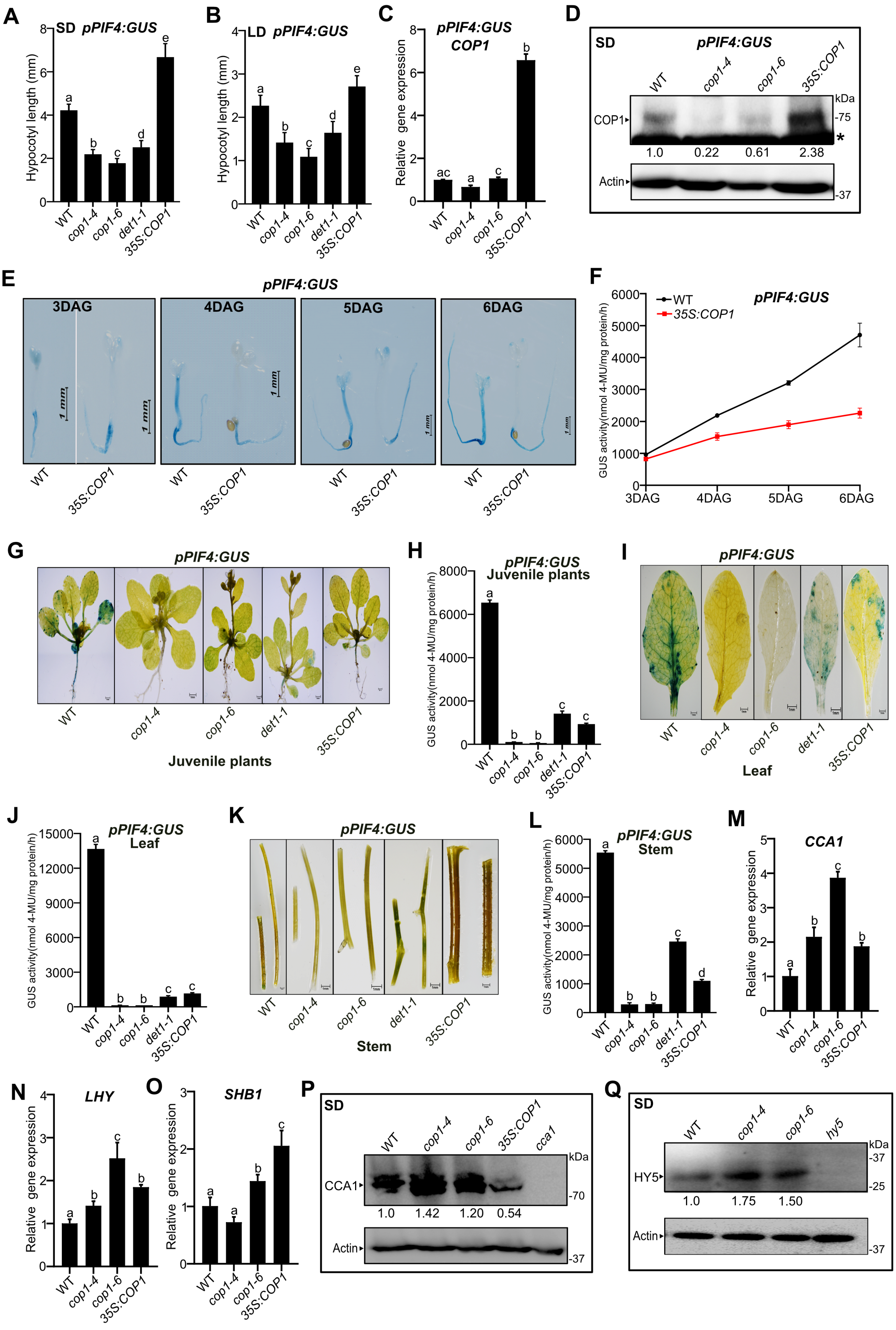
A
SD pPIF4:GUS
Hypocotyl length (mm)
WT
cop1-4
cop1-6
det1-1
35S:COP1
B
LD pPIF4:GUS
Hypocotyl length (mm)
C
pPIF4:GUS
COP1
Relative gene expression
D
SD
pPIF4:GUS
COP1
Actin
kDa
-75
-37
1.0
0.22
0.61
2.38
E
pPIF4:GUS
3DAG
4DAG
5DAG
6DAG
1 mm
F
pPIF4:GUS
WT
35S:COP1
GUS activity(nmol 4-MU/mg protein/h)
G
pPIF4:GUS
Juvenile plants
H
pPIF4:GUS
Juvenile plants
GUS activity(nmol 4-MU/mg protein/h)
I
pPIF4:GUS
Leaf
J
pPIF4:GUS
Leaf
GUS activity(nmol 4-MU/mg protein/h)
K
pPIF4:GUS
Stem
L
pPIF4:GUS
Stem
GUS activity(nmol 4-MU/mg protein/h)
M
CCA1
Relative gene expression
N
LHY
Relative gene expression
O
SHB1
Relative gene expression
P
SD
CCA1
Actin
cca1
kDa
-70
-37
1.0
1.42
1.20
0.54
Q
SD
HY5
Actin
hy5
kDa
-37
-25
-37
1.0
1.75
1.50

S4 Fig. COP1/DET1 promotes PIF4 autoinhibition.

(A and B) Hypocotyl length of six-day-old WT, *cop1-4, cop1-6, det1-1* and *35S:COP1* seedlings carrying the transgene *pPIF4:GUS* grown in SD (A) and LD (B) at 22°C.

(C and D) COP1 expression level of six-day-old WT, *cop1-4, cop1-6, det1-1* and *35S:COP1* seedlings carrying the transgene *pPIF4:GUS* grown in SD (ZT23) at 22°C. The *COP1* transcript level was measured through RT-qPCR (C), and immunoblots showed COP1 protein levels using native COP1 antibody (D). Values below the blots indicate fold-change compared to WT. Actin was used as the loading control. The asterisk (*) denotes a non-specific band.

(E and F) Day-wise GUS staining and GUS activity measurement from three-day-old (3-DAG) to six-day-old (6-DAG) WT and *35S:COP1* seedlings carrying the transgene *pPIF4:GUS* grown under SD. The tissue was collected at the ZT23 time point.

(G-L) GUS staining and GUS activity from the above-mentioned genotypes are detected in three-week-old juvenile plants (G and H), rosette leaves (I and J) and stems (K and L) of six-week-old adult plants grown at 22ºC under LD. Whole plant tissue was harvested for staining and activity at ZT4 time point.

(M-O) Gene expression of *CCA1* (M), *LHY* (N) and *SHB1* (O) from six-day-old WT, *cop1-4, cop1-6* and *35S:COP1*, grown under SD (22ºC). The tissue was collected at the ZT23 time point. *EF1α* was used as a housekeeping control.

(P and Q) Immunoblots of CCA1 (P) and HY5 (Q) using their respective native antibodies from six-day-old seedlings of the indicated genotypes grown under SD. The seedlings were harvested at ZT23. Values below the blots indicate fold-change compared to WT. Actin was used as the loading control. The *cca1* and *hy5* mutants were used as negative controls.

Data represent mean±SD; n>20 for the Hypocotyl length experiment and n=6 for the adult plant histochemical assay. For gene expression studies, the transcript level of each sample was normalized to that of WT. Error bars depict the mean ± SD of three biological replicates. Different letters indicate significant differences (one-way ANOVA with Tukey's HSD test, $P < 0.05$). The experiment was repeated thrice, and similar results were obtained. Related to Fig 3.
