# Supplementary material for: Threshold-dependent negative autoregulation of PIF4 gene expression optimizes growth and fitness in Arabidopsis
Source: PLoS Genet. 2025 Aug 11;21(8):e1011758. doi: 10.1371/journal.pgen.1011758 (PMC12338842; doi:10.1371/journal.pgen.1011758)
Supplement: S5 Fig — (PDF) [file pgen.1011758.s005.pdf]

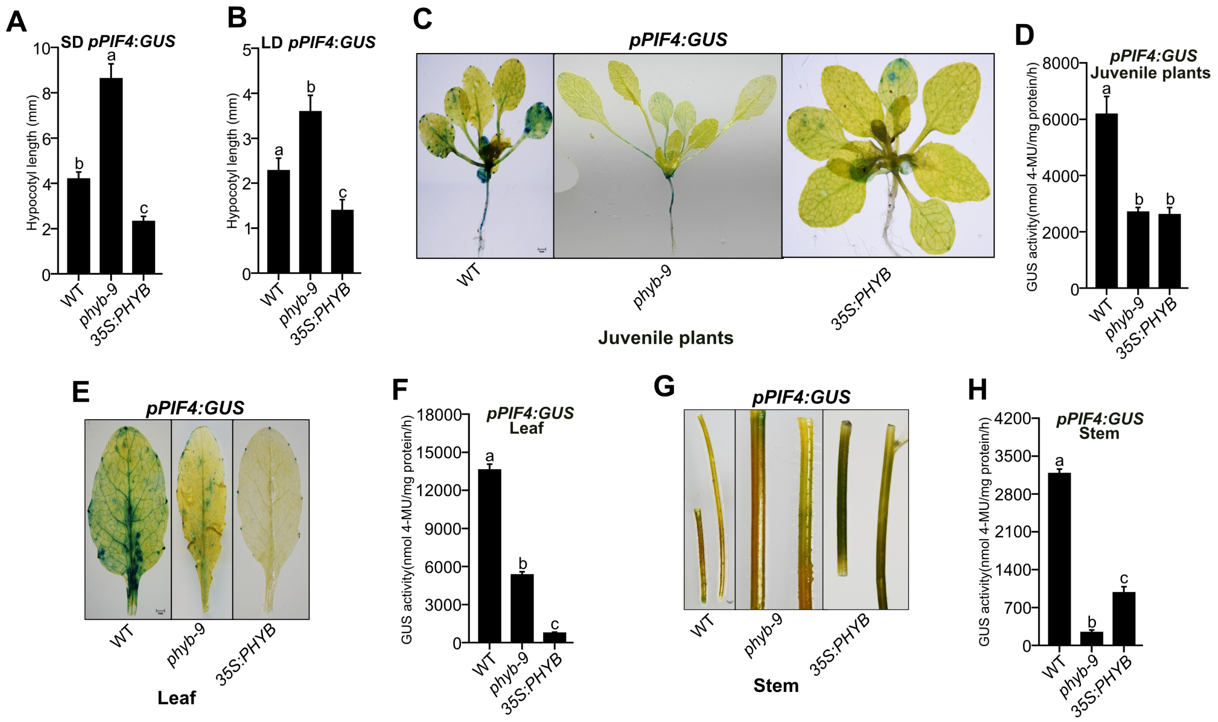

S5 Fig. The phyB photoreceptor inhibits PIF4 autoinhibition.

(A and B) Hypocotyl length of six-day-old WT, *phyb-9* and *35S:PHYB* seedlings carrying the transgene *pPIF4:GUS* grown in SD (A) and LD (B) at 22ºC.

(C-H) Representative GUS staining and GUS activity from three-week-old juvenile plants (C and D) and rosette leaves (E and F), stem (G and H) of six-week-old adult plants grown at 22ºC under LD. Whole plant tissue was harvested at ZT4. Data represent mean±SD; n>20 for the Hypocotyl length experiment and n=6 for the adult plant histochemical assay. Different letters indicate significant differences (one-way ANOVA with Tukey's HSD test, $P < 0.05$). The experiment was repeated thrice, and similar results were obtained. Related to Fig 4.
