# Supplementary material for: Threshold-dependent negative autoregulation of PIF4 gene expression optimizes growth and fitness in Arabidopsis
Source: PLoS Genet. 2025 Aug 11;21(8):e1011758. doi: 10.1371/journal.pgen.1011758 (PMC12338842; doi:10.1371/journal.pgen.1011758)
Supplement: S6 Fig — (PDF) [file pgen.1011758.s006.pdf]

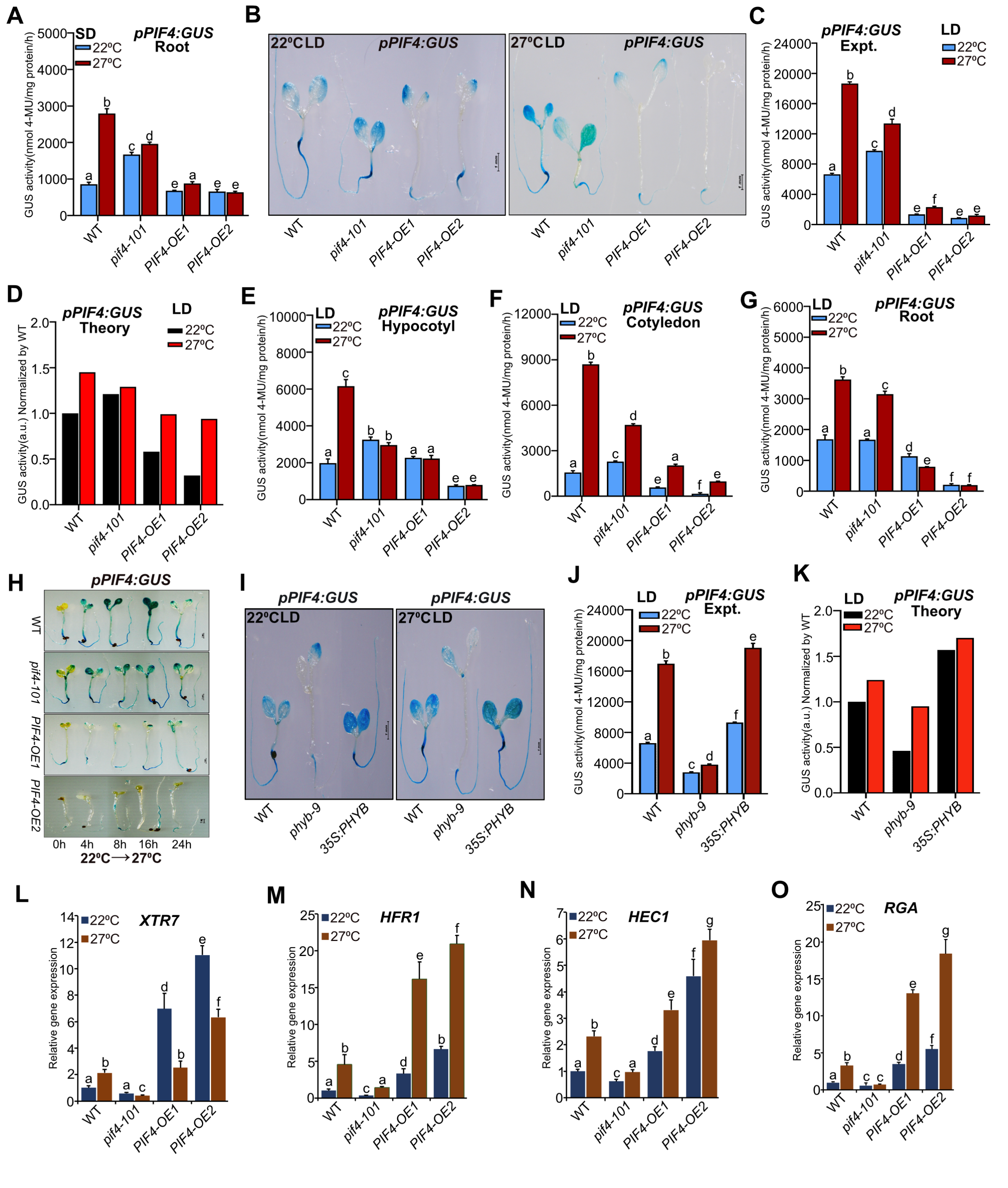

S6 Fig. Temperature-mediated autoinhibition of *PIF4* promoter activity depends on endogenous PIF4 protein concentration.

(A) GUS activities from the excised roots of six-day-old whole seedlings grown under SD at 22°C and 27°C (ZT23).

(B-D) Representative GUS-stained image (B), experimental GUS activity (C), and model-predicted GUS activity (D) of six-day-old seedlings grown under LD in WL at 22°C and 27°C (ZT4).

(E-G) Tissue-specific GUS activities measured from hypocotyl (E), cotyledon (F) and roots (G) of WT, *pif4-101*, *PIF4-OE1* and *PIF4-OE2* seedlings grown under 22°C and 27°C in LD (ZT4).

(H) Representative GUS-stained images of WT, *PIF4-OE1* and *PIF4-OE2* grown under 22°C for five days and shifted to 27°C for the mentioned time point under SD.
(I-K) Representative GUS-stained images of WT, *phyb9,* and *35S:PHYB* carrying the transgene *pPIF4:GUS* are shown (I), experimental GUS activity (J) and model-predicted GUS activity (K) measurement under 22°C and 27°C in LD at WL (ZT4).
(L-O)) Gene expression of *XTR7* (L), *HFR1* (M), *HEC1* (N) and *RGA* (O) at ZT23 as measured by qPCR in respective genotypes of six-days-old seedlings grown under SD in WL at 22°C and 27°C. *XTR7* is a growth-related gene, whereas *HFR1*, *HEC1* and *RGA* are growth-inhibiting genes. Data represent mean ± SD (n=3 biological replicates).
For GUS staining and activity measurement, the samples were harvested at ZT23 for SD and ZT4 for LD conditions. Data represent mean±SD. Different letters indicate significant differences (two-way ANOVA with Tukey's HSD test, $P < 0.05$). The experiment was repeated three times with similar results. Related to Figs 5 and 7.
