# Supplementary material for: Threshold-dependent negative autoregulation of PIF4 gene expression optimizes growth and fitness in Arabidopsis
Source: PLoS Genet. 2025 Aug 11;21(8):e1011758. doi: 10.1371/journal.pgen.1011758 (PMC12338842; doi:10.1371/journal.pgen.1011758)
Supplement: S7 Fig — (PDF) [file pgen.1011758.s007.pdf]

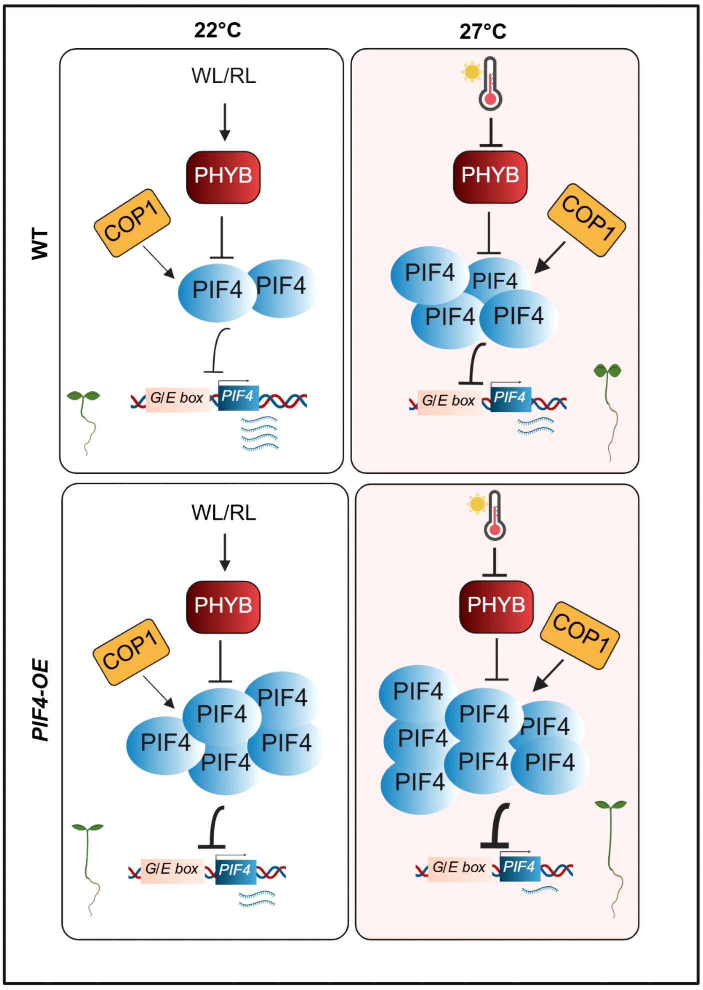

S7 Fig. Hypothetical model depicting the PIF4-mediated autoinhibition.
The proposed model illustrates that light (WL/RL) and warm temperature regulate PIF4 accumulation and autoinhibition of its own gene expression, depending on endogenous PIF4 concentration. In WT, under normal conditions (light, 22°C), the PIF4 protein accumulation and the corresponding autoinhibition are moderate and increased in response to warm temperature (27°C), whereas more PIF4 protein accumulated in PIF4-overexpressor (*PIF4-OE*) leads to strong autoinhibition under both the condition (22°C and 27°C). The extent of autoinhibition is indicated by the thickness of the inhibitory arrow. Compared to WT, the autoinhibition in *PIF4-OE* is consistently more pronounced. Upstream regulators, including phyB and COP1, are depicted; COP1 facilitates PIF4 accumulation in response to warm temperatures. The phenotypic outcome of this regulatory network is reflected in hypocotyl elongation observed in WT and *PIF4-OE* backgrounds. Created in BioRender. Das, S. (2025) https://BioRender.com/hylsmbm.
