# Supplementary material for: Threshold-dependent negative autoregulation of PIF4 gene expression optimizes growth and fitness in Arabidopsis
Source: PLoS Genet. 2025 Aug 11;21(8):e1011758. doi: 10.1371/journal.pgen.1011758 (PMC12338842; doi:10.1371/journal.pgen.1011758)
Supplement: S1 Table — The values are taken from [68], and they are fixed for all genotypes. Note that the shaded boxes denote the parameters that slightly change from 22°C to 27°C. (PDF) [file pgen.1011758.s009.pdf]

Table S1. The description of parameters and corresponding values. The values are taken from [1], and they are fixed for all genotypes. Note that the shaded boxes denote the parameters that slightly change from $22^{o}\mathrm{C}$ to $27^{o}\mathrm{C}$.

Description of parameters	Values at $22^{o}\mathrm{C}$	Values at $27^{o}\mathrm{C}$
Light-induced activation rate of phyB	$p_B = 10.0$	$p_B = 0.860$
Deactivation rate of phyB in dark	$k_r = 0.232$	$k_r = 0.411$
Parameter related to the production rate of ELF3 (see Equation 2)	$p_{E1} = 108$	$p_{E1} = 127$
Parameter related to the production rate of ELF3 (see Equation 2)	$p_{E2} = 39.8$	$p_{E2} = 7.29$
Production rate of COP1 in light	$p_{CL} = 1.00$	$p_{CL} = 5.37$
Intensity of ELF3's inhibition of PIF4 production	$p_{PE} = 0.332$	$p_{PE} = 0.028$
Production rate of COP1 in dark	$p_{CD} = 112$	$p_{CD} = 112$
Decay rate of ELF3	$d_E = 27.2$	$d_E = 27.2$
Production rate of hypocotyl growth	$p_{GP} = 2.93$	$p_{GP} = 2.93$
Decay rate of COP1	$d_C = 1.79$	$d_C = 1.79$
Decay rate of PIF4	$d_P = 4.91$	$d_P = 4.91$
Basal rate of hypocotyl growth	$p_G = 0.009$	$p_G = 0.009$
Intensity of phyB's inhibition of hypocotyl growth	$p_{GB} = 10.7$	$p_{GB} = 10.7$
Inhibition rate of PIF4 by phyB	$d_{PB} = 0.313$	$d_{PB} = 0.313$
Conversion factor between PIF4-targeted gene expression and hypocotyl growth	$k_G = 0.113$	$k_G = 0.113$
Intensity of ELF3's inhibition of hypocotyl growth	$p_{GE} = 0.465$	$p_{GE} = 0.465$
Intensity of COP1's inhibition of PIF4 degradation	$k_{PC} = 34.3$	$k_{PC} = 34.3$
Parameter defining the sharpness of transitions between maximum and minimum production rates of ELF3	$\alpha = 5$	$\alpha = 5$
Production rate of PIF4	$p_P = 1$	$p_P = 1$

Reference

1. Nieto C, Luengo LM, Prat S. Regulation of COP1 Function by Brassinosteroid Signaling. Front Plant Sci. 2020;11:1151. Epub 20200731. doi: 10.3389/fpls.2020.01151. PubMed PMID: 32849709; PubMed Central PMCID: PMCPMC7411146.
