# Supplementary material for: Threshold-dependent negative autoregulation of PIF4 gene expression optimizes growth and fitness in Arabidopsis
Source: PLoS Genet. 2025 Aug 11;21(8):e1011758. doi: 10.1371/journal.pgen.1011758 (PMC12338842; doi:10.1371/journal.pgen.1011758)
Supplement: S2 Table — Note that the shaded boxes represent the parameter values obtained by fitting to the wildtype data and kept unchanged for all other genotypes. (PDF) [file pgen.1011758.s010.pdf]

S2 Table: Parameter values estimated (with uncertainty) from non-linear least squares fitting. Note that the shaded boxes represent the parameter values obtained by fitting to the wildtype data and kept unchanged for all other genotypes.

Genotypes	Description of parameters	Values at $22^{o}\mathrm{C}$ (Estimated)	Values at $27^{o}\mathrm{C}$ (Assumed)
**WT**	Inhibition rate of ELF3 by COP1	$d_{EC} = 0.01 \pm 0.0133$	$d_{EC} = 0.01$
	Basal rate of GUS production	$k_0 = 10 \pm 0.2007$	$k_0 = 10$
	Production rate of GUS	$p_F = 450 \pm 10.7729$	$p_F = 450$
	Decay rate of GUS	$d_F = 0.0009 \pm 0.0206$	$d_F = 0.0009$
	Intensity of PIF4's inhibition of GUS expression	$p_{FP} = 22 \pm 0.0.3975$	$p_{FP} = 22$
	Multiplier ($mut_k = 1; k = E, B, P, C$)	$mut_k = 1$	$mut_k = 1$
	PIF4 threshold concentration	$P^* = 0.2 \pm 0.00001$	$P^* = 0.2$
	Negative feedback strength	$p_{self} = 25 \pm 0.3534$	$p_{self} = 20$
pif4-101	Multiplier	$mut_P = 0.65 \pm 0.0214$	$mut_P = 0.65$
	PIF4 threshold concentration	$P^* = 0.04 \pm 0.0242$	$P^* = 0.04$
	Negative feedback strength	$p_{self} = 25 \pm 0.5818$	$p_{self} = 5$
PIF4-OE1	Multiplier	$mut_P = 4 \pm 1.0599\mathrm{E}-09$	$mut_P = 4$
	PIF4 threshold concentration	$P^* = 0.2 \pm 1.58\mathrm{E}-09$	$P^* = 0.2$
	Negative feedback strength	$p_{self} = 25 \pm 0.00031$	$p_{self} = 20$
PIF4-OE2	Multiplier	$mut_P = 5.9 \pm 0.0000001$	$mut_P = 5.9$
	PIF4 threshold concentration	$P^* = 1.3 \pm 3.88\mathrm{E}-08$	$P^* = 1.3$
	Negative feedback strength	$p_{self} = 25 \pm 4.350\mathrm{E}-07$	$p_{self} = 20$
phyb-9	Multiplier	$mut_B = 0.6 \pm 0.000021$	$mut_B = 0.6$
	PIF4 threshold concentration	$P^* = 0.6 \pm 2.07\mathrm{E}-09$	$P^* = 0.6$
	Negative feedback strength	$p_{self} = 25 \pm 3.021\mathrm{E}-07$	$p_{self} = 5$
35S:PHYB	Multiplier	$mut_B = 4 \pm 0.0585$	$mut_B = 4$
	PIF4 threshold concentration	$P^* = 0.1 \pm 0.0136$	$P^* = 0.1$
	Negative feedback strength	$p_{self} = 25 \pm 0.5215$	$p_{self} = 23$
cop1-4	Multiplier	$mut_C = 0.001 \pm 0.00006$	-------
	PIF4 threshold concentration	$P^* = 0.35 \pm 2.213\mathrm{E}-20$	-------
	Negative feedback strength	$p_{self} = 25 \pm 1.48\mathrm{E}-07$	-------
35S:COP1	Multiplier	$mut_C = 50 \pm 0.61114$	-------
	PIF4 threshold concentration	$P^* = 0.6 \pm 0.01429$	-------
	Negative feedback strength	$p_{self} = 25 \pm 0.32414$	-------
