# Supplementary material for: Threshold-dependent negative autoregulation of PIF4 gene expression optimizes growth and fitness in Arabidopsis
Source: PLoS Genet. 2025 Aug 11;21(8):e1011758. doi: 10.1371/journal.pgen.1011758 (PMC12338842; doi:10.1371/journal.pgen.1011758)
Supplement: S3 Table — Shaded boxes represent the unchanged parameter values for all genotypes. (PDF) [file pgen.1011758.s011.pdf]

S3 Table: Parameter values to produce the dynamics of PIF4 protein and GUS activity (corresponding to Figures 6G and 6H). Shaded boxes represent the unchanged parameter values for all genotypes.

Genotypes	Description of parameters	Values at 22 Degree	Values at 27 Degree
**WT**	Light-induced activation rate of phyB	$P_B = 10$	$P_B = 5$
	Deactivation rate of phyB in dark	$k_r = 0.232$	$k_r = 0.251$
	Production rate of COP1 in light	$p_{CL} = 1$	$p_{CL} = 2.37$
	Basal rate of GUS production	$k_0 = 10$	$k_0 = 100$
	Negative Feedback strength at the beginning of the day	$P_{self}^{max/day} = 70$	$P_{self}^{max/day} = 20$
	Negative feedback strength in the midday	$P_{self}^{min} = 0.1$	$P_{self}^{min} = 0.1$
	Negative feedback strength at night	$P_{self}^{max/night} = 80$	$P_{self}^{max/night} = 20$
	PIF4-mediated GUS inhibition rate at the beginning of the day	$P_{FP}^{max/day} = 30$	$P_{FP}^{max/day} = 5$
	PIF4-mediated GUS inhibition rate in the midday	$P_{FP}^{min} = 0.5$	$P_{FP}^{min} = 0$
	PIF4-mediated GUS inhibition rate at night	$P_{FP}^{max/night} = 10$	$P_{FP}^{max/night} = 5$
PIF4-OE2	Basal rate of GUS production	$k_0 = 1$	$k_0 = 20$
	Negative Feedback strength at the beginning of the day	$P_{self}^{max/day} = 90$	$P_{self}^{max/day} = 85$
	Negative feedback strength in the midday	$P_{self}^{min} = 1$	$P_{self}^{min} = 0.95$
	Negative feedback strength at night	$P_{self}^{max/night} = 110$	$P_{self}^{max/night} = 100s$
	PIF4-mediated GUS inhibition rate at the beginning of the day	$P_{FP}^{max/day} = 300$	$P_{FP}^{max/day} = 300$
	PIF4-mediated GUS inhibition rate in the midday	$P_{FP}^{min} = 50$	$P_{FP}^{min} = 30$
	PIF4-mediated GUS inhibition rate at night	$P_{FP}^{max/night} = 300$	$P_{FP}^{max/night} = 300$
