# Supplementary material for: Threshold-dependent negative autoregulation of PIF4 gene expression optimizes growth and fitness in Arabidopsis
Source: PLoS Genet. 2025 Aug 11;21(8):e1011758. doi: 10.1371/journal.pgen.1011758 (PMC12338842; doi:10.1371/journal.pgen.1011758)
Supplement: S4 Table — (PDF) [file pgen.1011758.s012.pdf]

S4 Table. List of oligonucleotides and their sequences used in the study.

Name	Oligo no.	Oligo Sequence (5'…………3')	Purpose
**Primers used for genotyping PCR analysis**			
pif4-101-LP	290	AATACATTTTGCAGGCAATCG	Genotyping pif4-101
pif4-101-RP	291	CGTAATGAAGTTGCACGTTTACTC	Genotyping pif4-101
pif4-101-LB (SAIL)	185	TTTCATAACCAATCTCGATACAC	T-DNA specific oligo for genotyping pif4-101
phyb-9-FP	542	CAATGTAGCTAGTGGAAGAAGCTCGATGTGG	Genotyping cut PCR-product with MnlI to distinguish between mutant and WT
phyb-9-RP	543	ACATAACAGTGTCTGCGTTCTCAAAACGC	Genotyping
LUC-FP	616	ACACCCGAGGGGGATGATAA	Genotyping
LUC-RP	617	GGCGACGTAATCCACGATCT	Genotyping
**Primers used for cloning purposes**			
PIF4-PRO-FP	760	CACCTTAACCGCCGCAACAAGTC	Gateway cloning
PIF4-PRO-RP	761	GTCGATACCTTTCTCATATATACC	Gateway cloning
PIF4-AD-Nde1-FP	13	GGGAATTCCATATGGAACACCAAGGTTGGAGTTTTGAGGAG	Y2H cloning
PIF4-AD-Xho1-RP	14	CCGCTCGAGCTAGTGGTCCAAACGAGAACCGTC	Y2H cloning
Promoter PIF4-FP-HindIII	639	CCCAAGCTTATGTTTCCCAAACACATTTGGGGCCG	Y1H cloning
Promoter PIF4-RP-KpnI	645	CGGGGTACCCATAAAGATATTACAGCGAGGGAGAT	Y1H cloning
**Primers used for gene expression analysis**			
PIF4-FP	512	ACCTCAGAGACGGTTAAGCC	qPCR
PIF4-RP	513	TGGAGGAGGCATGACTTGAG	qPCR
YUC8-FP	508	CGTCTCAAGCTTCACCTTCC	qPCR
YUC8-RP	509	AGCCACTGGTCTCATCGAAC	qPCR
5'UTR+2ND EXON-PIF4-FP	673	GGGCGTGGAACTTGGACTA	qPCR
5'UTR+2ND EXON-PIF4-RP	674	CCACTTGTCCATCTCGCCATA	qPCR

IAA29-FP	798	TCCGATTTGAACGCCTATCCT	qPCR
IAA29-RP	799	ACCGTGTGCATATACAAGATGTTTG	qPCR
XTR7-FP	510	CGGCTTGCACAGCCTCTT	qPCR
XTR7-RP	511	TCGGTTGCCACTTGCAATT	qPCR
HEC1-FP	1315	GAGGAAGGGTTTTGATCGGTGGAG	qPCR
HEC1-RP	1316	TGCATTGCCCACCATCTGATGAGT	qPCR
HFR1-FP	1311	GATGCGTAAGCTACAGCAACTCGT	qPCR
HFR1-RP	1312	AGAACCGAAACCTTGTCCGTCTTG	qPCR
RGA-FP	1349	CATTCCCGGAAACGCGATTTATCAG	qPCR
RGA-RP	1350	TCACCGTCGTTCCTATGACTCCA	qPCR
COP1-FP	840	AAGAGTGTAGTACGGAGGGAAGG	qPCR
COP1-RP	841	TAGACGACTGTAGCGAGTGAAGG	qPCR
CCA1-FP	268	GATCTGGTTATTAAGACTCGGAAGCC ATATAC	qPCR
CCA1-RP	269	GCCTCTTTCTCTACCTTGGAGA	qPCR
LHY-FP	270	AAGTCTCCGAAGAGGGTC	qPCR
LHY-RP	271	ATGTTCCAACACCGATC	qPCR
SHB1-FP	392	AAGAATGGTGGAAGACAGAGAT	qPCR
SHB1-RP	393	AGAAGCAGCAACGATGGT	qPCR
GUS-FP	1455	CCCTTACGCTGAAGAGATGC	qPCR
GUS-RP	1456	GAGGTTAAAGCCGACAGCAG	qPCR
**Primers used for ChIP analysis**			
PIF4-FP (-401bp)	1648	CCATTCGTGTGAATGTTTCCCA	ChIP-qPCR
PIF4-RP (-401bp)	1649	TAGCCAAATACAGGACGCGGAAT	ChIP-qPCR
PIF4-FP (-651 bp)	1646	TGAAAATTTGAAATCCGTATGGTC	ChIP-qPCR
PIF4-RP (-651 bp)	1647	TAATGGACCATATCCCATACC	ChIP-qPCR
PIF4-FP (-160 bp)	643	CCCAAGCTTAGAGATCTTCACTTGTA TGTGTC	ChIP-qPCR
PIF4-RP (-160 bp)	645	CGGGGTACCCATAAAGATATTACAGC GAGGGAGAT	ChIP-qPCR
PIF4-FP (-113 bp)	644	CCCAAGCTTATGTCCCAGAACTTGCC ACGTGTCGTT	ChIP-qPCR

\
