# Supplementary material for: Threshold-dependent negative autoregulation of PIF4 gene expression optimizes growth and fitness in Arabidopsis
Source: PLoS Genet. 2025 Aug 11;21(8):e1011758. doi: 10.1371/journal.pgen.1011758 (PMC12338842; doi:10.1371/journal.pgen.1011758)
Supplement: S2 Fig — (PDF) [file pgen.1011758.s002.pdf]

Authors' Response to Reviewers' Comments PGENETICS-D-24-01351

Author response: Please see our responses in blue.

Prof. Enamul Haq
Guest Editor
PLOS Genetics,

Monica Colaiácovo
Section Editor
PLOS Genetics

Aimée Dudley
Editor-in-Chief
PLOS Genetics

Anne Goriely
Editor-in-Chief
PLOS Genetics

Dear Editors,

We are submitting a revised version of the manuscript (**PGENETICS-D-24-01351)** titled 'Threshold-dependent negative autoregulation of PIF4 gene expression optimizes growth and fitness in Arabidopsis', that we have submitted for publication in ***PLOS Genetics*** [EMID:65cabdb9546437e9]

Thank you for giving us the opportunity to revise and resubmit our manuscript. We have revised our manuscript according to the Reviewer's suggestions and addressed all the concerns with additional data and discussion. Please find our point-by-point response to the Reviewers' comments. Also, please find our specific comments on the copyright-related issues below. We hope the revised version of the manuscript now fully meets PLOS Genetics' publication criteria.

We are looking forward to hearing a positive decision soon.

Sincerely,

Sreeramaiah N. Gangappa and Dipjyoti Das on behalf of all co-authors.

Authors' Response to Reviewers' Comments PGENETICS-D-24-01351

Journal Requirements:

1) We ask that a manuscript source file is provided at Revision. Please upload your manuscript file as a .doc, .docx, .rtf or .tex. If you are providing a .tex file, please upload it under the item type u2018LaTeX Source Fileu2019 and leave your .pdf version as the item type u2018Manuscriptu2019.

Author response: We have provided the manuscript text file.

2) We do not publish any copyright or trademark symbols that usually accompany proprietary names, eg ©, ®, or TM (e.g. next to drug or reagent names). Therefore please remove all instances of trademark/copyright symbols throughout the text, including:
- TM on pages: 26, and 27.

Author response: We have removed all the copyright or trademark symbols.

3) Please upload all main figures as separate Figure files in .tif or .eps format. For more information about how to convert and format your figure files please see our guidelines: https://journals.plos.org/plosgenetics/s/figures

Author response: We have uploaded the main figures in .tif file format.

4) We have noticed that you cited Figure S6I and S6J on page 18 in your manuscript. However, there is no corresponding file uploaded to the submission. Please include it in the Supporting Information file or remove all reference to it within the text.

Author response: Thank you for informing me about this discrepancy. We have included it in the revised manuscript.

5) Some material included in your submission may be copyrighted. According to PLOSu2019s copyright policy, authors who use figures or other material (e.g., graphics, clipart, maps) from another author or copyright holder must demonstrate or obtain permission to publish this material under the Creative Commons Attribution 4.0 International (CC BY 4.0) License used by PLOS journals. Please closely review the details of PLOSu2019s copyright requirements here: PLOS Licenses and Copyright. If you need to request permissions from a copyright holder, you may use PLOS's Copyright Content Permission form.

Please respond directly to this email and provide any known details concerning your material's license terms and permissions required for reuse, even if you have not yet obtained copyright permissions or are unsure of your material's copyright compatibility. Once you have responded and addressed all other outstanding technical requirements, you may resubmit your manuscript within Editorial Manager.

Author response: We have carefully checked our manuscript and confirmed that all figures and associated materials have been created by us. The graphics used in Figure 2A were designed by Krishanu Mondal, author of this manuscript, and none of the graphics require copyright permission. The working model_illustrations presented in Figures 7 and S7 were created using BioRender, and the corresponding publication licence file has been attached, which is under the CC BY 4.0 Licence. Apart from these, no external materials requiring copyright permission have been used in the manuscript.

Authors' Response to Reviewers' Comments PGENETICS-D-24-01351

Potential Copyright Issues:

i) Please confirm (a) that you are the photographer of 1C, 1F, 3A, 3F, 3K, 3L, 4A, 4F, 4J, , 5A, 5H, S1C, S1E, S1Q, S2C, S2E, S3A, S3C, S3E, S3G, S4D, S4F, S4H, S4L, S4N, S4P, S5B, S5H, and S5I, or (b) provide written permission from the photographer to publish the photo(s) under our CC BY 4.0 license.

Author response: Vikas Garhwal and Sreya Das lead authors of the paper, have taken all the photographs of the following images 1C, 1F, 3A, 3F, 3K, 3L, 4A, 4F, 4J, 5A, 5H, S1C, S1E, S1Q, S2C, S2E, S3A, S3C, S3E, S3G, S3I S4D, S4E, S4G, S4I, S4K, S5C, S5E, S5G, S6B, S6H and S6I. S3I and S4E are the new images added in the revised manuscript.

The author has a premium account for BioRender and uploaded a pdf of the publication licence of the CC BY 4.0 Licence as provided by BioRender. The Fig. 7 (Created in BioRender. Das, S. (2025) https://BioRender.com/zxysze4) and Fig. S7 (Created in BioRender. Das, S. (2025) https://BioRender.com/hylsmbm) were created in Biorender with unique figure URL. We have obtained the publication license from Biorender for the same. We have sent these licenses by mail, and also along with the manuscript.

6) Please amend your detailed Financial Disclosure statement. This is published with the article. It must therefore be completed in full sentences and contain the exact wording you wish to be published.

If the funders had no role in your study, please state: "The funders had no role in study design, data collection and analysis, decision to publish, or preparation of the manuscript."

Author response:

Financial Disclosure Statement: This work is supported by the Govt. of India grants from the Department of Biotechnology (Ramalingaswami Re-entry Fellowship grant, BT/RLF/Re-entry/ 28/2017), Science and Engineering Research Board (start-up research grant, SRG/2019/000446), Anusandhan National Research Foundation (Core Research Grant, CRG/2023/001553), and an intramural grant from IISER Kolkata to S.N.G. Research from D.D. lab is supported by the Science and Engineering Research Board, Govt. of India (EEQ/2023/000551). The funders had no role in study design, data collection and analysis, decision to publish, or preparation of the manuscript."

Please note that we have added the above-revised paragraph in the manuscript as well.

7) Please ensure that the funders and grant numbers match between the Financial Disclosure field and the Funding Information tab in your submission form. Note that the funders must be provided in the same order in both places as well. Currently, "IISER Kolkata Intramural grant (Ministry of Education, Government of India)" is missing from the Funding Information tab.

Author response: Thank you for this. Now, we have corrected this.

Authors' Response to Reviewers' Comments PGENETICS-D-24-01351

Response to Reviewer's Comments:

Author response: Dear Reviewers, we sincerely thank you for your time and efforts in evaluating our manuscript. We appreciate your overall positive outlook on our manuscript and the valuable comments and suggestions you provided. We have conducted additional experiments, the results of which further reinforce our primary conclusion and enhance the manuscript's quality. Below, we have addressed each of your comments with detailed, point-by-point responses. The revised manuscript includes edits highlighted in yellow for your convenience. We believe these revisions address your concerns and hope the updated manuscript meets the standards for publication in *PLoS Genetics*.

Reviewer #1:

This study by Das et al., report that PIF4 undergoes a threshold-dependent autoinhibition of itself at the transcriptional level. This regulatory event contributes to the growth and biomass accumulation in Arabidopsis. Although previous studies have shown that PIF4 binds to its own promoter regions to control its transcription, the detailed molecular mechanism and biological significance are not clear. Overall, this is a nice story. The conclusions claimed in this study are well-supported by the genetic and biochemical data. I only have a few suggestions.

Author response: We thank the reviewer for the insightful comments, which helped us substantially improve the manuscript's quality. Please see below our point-by-point response.

1. Please indicate (where it is missing) how many biological replicates were used in each of the genetic and biochemical studies.

Author response: Thank you for your suggestion. We have mentioned the biological replicates in the figure legend and the section "Sampling of biological samples for various assays" (Materials and Methods). At least 20 seedlings were used to measure hypocotyl length. For GUS staining, ~20-25 seedlings were harvested and stained. The stained representative seedlings were used for the reference images. For GUS activity measurement, tissue was harvested in two biological replicates, and three technical replicates were used. For gene expression analysis, three biological replicates were sampled at specific time points. For Western blots, at least three biological replicates were used.

2. The working model showing in Figure 7G lacking the light (red light) and warm temperatures-regulated PIF4 accumulation and autoinhibition of its own expression.

Author response: Thank you for the suggestion. However, we kept only the effects of COP1 and phyB on PIF4 autoinhibition under white light since our study thoroughly investigated and established these conclusions. Though we also present some data on the effects of red light and warm temperature, a more detailed analysis will be required in the future to build a holistic model. Nevertheless, we propose a hypothetical model that includes red light and warm temperature in the Supplementary Information (Figure S7), while we keep the main model unchanged (Figure 7G).

3. Please indicate the number of plants were used to measure the rosette biomass (Figure 7C-D).

Author response: We have used six plants to measure the rosette biomass, which has been mentioned in the legend of Figure 7 in the revised manuscript.

Reviewer #2:

PIF4 is a central transcription factor integrating various environmental signals, including light and temperature, to regulate plant growth and development. Previous studies have shown that PIF4 can bind to its own promoter, indicating a feedback mechanism regulating its own transcription. In this manuscript, Das et al. propose an interesting model in which PIF4's autoregulation is threshold-dependent and essential for maximising growth and fitness. I find this model quite compelling, and I believe that most of the data support it well. Below are my comments:

Author response: We thank the reviewer for the suggestion. Please see below our point-by-point response to your comments.

1. In the abstract and introduction, the authors state that the regulation of PIF4 transcription is poorly understood. However, recent studies by several groups have demonstrated that a number of transcription factors, including HSFA1, TCP5, and BBX18, regulate PIF4 transcription. Thus, the authors' statement is not entirely accurate and should be revised. These studies also need to be incorporated into the introduction and discussed accordingly.

Author response: We thank the reviewer for the suggestion. We have added some recent reports to our revised manuscript. We have included the following text in the introduction and also discussed it in the appropriate place wherever necessary.

"For instance, it has been discovered that HEMERA (HMR) stabilizes PIF4, regulates PIF4 target genes, and therefore controls thermomorphogenesis in Arabidopsis [1]. Heat shock transcription factors (HSFs), HSFA1s, bind to PIF4 and block its interaction with active phyB Pfr. PIF4 accumulation activates thermoresponsive genes, including *YUC8*, leading to increased daytime thermomorphogenesis [2]. Warm temperatures activate HSFA2 expression during the day through HSFA1 regulators, leading to heat shock reactions and thermotolerance [2, 3]. TCP5 directly interacts with PIF4 to enhance the stability and activity of the PIF4 protein, but it also directly binds the PIF4 promoter and increases the transcriptional level of PIF4 [4]. TCP5 and PIF4 coactivate numerous temperature-responsive genes to promote hypocotyl and petiole development [4]. Together with clock components CIRCADIAN CLOCK ASSOCIATED 1 (CCA1) and LATE ELONGATED HYPOCOTYL (LHY), the SHORT HYPOCOTYL UNDER BLUE1 (SHB1) directly binds to the *PIF4* promoter to activate its transcription [5]. The B-BOX (BBX) proteins BBX18 and BBX23 also regulate a subset of PIF4-dependent thermoresponsive genes by inhibiting EARLY FLOWERING 3 (ELF3) function, emphasizing the significance of BBX18 and BBX23 in thermomorphogenesis [6]."

Notably, most of the above studies have focused on PIF4 activation, while our study highlights the possible model of PIF4 autoinhibition.

2. The Western blot data appear rather messy. I can see multiple bands in the wild-type, whereas most of these bands are absent in the pif4 mutant. Does this imply that all of these bands represent PIF4? Is this situation typical?

Apart from these points, I have no further questions.

Author response: We appreciate your thoughts on this. The PIF4-specific bands come around 60 kDa, where we could not detect bands for the *pif4* mutant in the blots. The messy-looking bands for PIF4 are likely due to both phosphorylated and non-phosphorylated forms of PIF4, which could result in multiple bands corresponding to different modified forms of the protein, and we could not see those bands in the mutant and WT. We have repeated our western blots at least three times and observed similar results. A similar band pattern for PIF4 was also observed in an earlier report [7, 8].

Reviewer #3:

This study shows how PIF4 autoinhibits its own promoter activity in a concentration and photoperiod dependent manner. The authors have used both experimental analysis and mathematical modelling to try substantiating their hypothesis. They have used PIF4 mutant and overexpressor lines as well as different pPIF4-GUS lines in different genetic backgrounds for the study. They observed that PIF4 promoter activity is increased in pif4, whereas it is reduced in PIF4 overexpression lines. Authors also bring in the possible role of phyB and COP1 in mediating PIF4 autoinhibition. While the study is interesting, I have some reservations about the claims in the study which lack proper experimental evidence and some other comments that are given below.

Author response: We thank the reviewer for the insightful and valuable comments, which helped substantially improve the overall quality and the conclusions of this study. We have addressed your comments with additional experimental data and discussions. Please see below our point-by-point response to your comments.

Major Comments:

1. The authors have used the term 'threshold concentration' several times in the manuscript. What is the threshold concentration?

Author response: We propose that "the threshold concentration of PIF4" is the protein concentration above which the autoinhibition kicks in and induces lower promoter activity. In contrast, below the threshold, the *PIF4* promoter activity should be higher. For instance, in the *PHYB* overexpression line, the promoter activity is higher than wild type (WT), suggesting that the *PHYB* overexpression line is below the threshold (Figure 4A-D). However, the *phyb* mutant remains at a higher threshold and thus shows lesser *PIF4* activity than the WT. Similarly, the COP1 overexpression line behaves like the *phyb* mutant, showing lesser promoter activity than the WT and remaining at a higher threshold (Figure 3A-E). Interestingly, the *cop1* and *det1* mutants show strongly reduced promoter activity, suggesting that the COP1/DET1 module is required for a basal level of *PIF4* promoter activity and protein accumulation.

Though our data suggests the underlying existence of PIF4 threshold concentration for autoinhibition, this threshold may vary with photoperiod, temperature and other genetic perturbations. In line with this, a recent report suggests that PIF4 may even autoactivate below a threshold concentration [9]. However, the accurate estimation of such a

threshold can be done only at a single cell level by measuring protein and RNA concentrations. At a tissue level, the threshold would be variable due to environmental noise.

2. Page no: 13 "However, an exact quantitative comparison of the GUS activity with experimental data cannot be performed since our model's GUS activity is measured in arbitrary units." Please elaborate on how the GUS activity is measured using the mathematical model in "arbitrary units".

Author response: An "arbitrary unit" (a.u) refers to a non-standardised unit of measurement used for relative comparisons of some quantity. The value of an arbitrary unit is influenced by the measurement units of other variables and the chosen unit of time intervals for a measurement (like hours, minutes, etc.).

In our mathematical model, many variables, such as the diurnal dynamics of ELF3, COP1, and phyB concentrations, were not directly measured. Due to the lack of such information regarding the units of other variables, an exact quantitative comparison could not be performed. Rather, we aim to compare qualitatively the relative GUS activity with respect to wild-type values (such as Figure 2E-G). Nevertheless, such a convention of units was also used in previous theoretical models [10].

3. In Figure 1 b and d, according to authors, PIF4 expression is more in the overexpressor than wt but promoter activity is less. The overexpressor (pPIF4:PIF4-FLAG) authors used were under native promoter. Kindly explain this observation.

Author response: Even though the PIF4 is expressed under its own promoter, PIF4 is being overexpressed as evident from its enhanced growth phenotypes [11], which is also due to increased PIF4 protein accumulation compared to Col-0 (WT) as seen in the immunoblot analysis (Figure 1A). Thus, we wanted to use these overexpression lines (*PIF4-OE1* and *PIF4-OE2*) to study the effect of PIF4 overexpression on its promoter activity using *pPIF4:GUS* promoter-reporter transgenic line. In the *PIF4-OE* lines, the *pPIF4:GUS* promoter activity was strongly reduced compared to the Col-0 background, likely due to negative autoregulation resulting from elevated PIF4 protein accumulation (Figure 1).

4. Figure 1 n, authors showed the GUS activity in red light, one experiment with gus activity comparison between red light and white light in wt is needed here just to show that what is the effect of red light on PIF4 promoter activity in comparison to white light.

Author response: Thank you for the suggestion. We analysed GUS activity under white light (WL) and red light (RL) and found that *PIF4* promoter activity was significantly higher in RL than in WL Figure S3I and S3J). However, in the PIF4 overexpression lines, the GUS activity is significantly lower than wild-type under RL and WL conditions, suggesting that the autoinhibition is present under both RL and WL.

5. Reason for PIF4 promoter activity reduction in pif4-101 mutant under red light is not explained properly.

Author response: The most likely reason for reduced *PIF4* promoter activity in *pif4-101* mutant under red light (RL) is stronger negative feedback in RL than in white light (WL). We also simulated the WL and RL conditions in our mathematical model by increasing the strength of autoinhibition in RL compared to WL. This assumption was

sufficient to qualitatively reproduce the observed differential GUS activity in WL and RL (compare Figures 1D, 1N and Figures 2E, 2F). Moreover, please note that the relative level of *PIF4* mRNA in the overexpression lines under RL is lower than the WL as compared to the respective wild-type (Figures 1B and 1P), again suggesting stronger negative feedback in RL than in WL.

6. What is the rationale behind choosing ZT23 as the timepoint for harvesting seedlings for the experiments?

Author response: Thank you for the comment. Earlier reports suggest that PIF4 accumulation peaks at ZT23 under 22°C in short-day photoperiod and promotes the expression of growth-related genes and subsequently hypocotyl growth [12-16]. As we are interested in checking the effect of increased PIF4 levels on *PIF4* gene expression and promoter activity, we chose ZT23 for the short-day grown seedlings in the experiments.

7. Under SD condition, at ZT23, when PIF4 mRNA, protein and its activity all are at the peak to promote hypocotyl elongation, then it needs a better experimental clarification why PIF4OE1 (which is driven by PIF4 promoter) showing higher transcript level but the introgressed line with pPIF4:GUS showing reduced promoter activity compared to wild-type. Kindly explain.

Author response: We thank the reviewer for the insightful comments. We want to emphasize that we cannot always expect a linear one-to-one correspondence between the PIF4 protein and its transcript levels in the presence of negative feedback. For instance, *35S:PHYB* overexpression has reduced PIF4 protein levels and hence weaker autoinhibition, leading to higher promoter activities and higher transcript levels than WT (Figures 4D). On the other hand, *PIF4-OE2* has significantly more PIF4 protein and stronger autoinhibition, resulting in lower promoter activities and or comparable transcript levels to WT (Figure 1A and 1B). However, when the PIF4 protein levels are moderately higher than WT, as in the cases of *PIF4-OE1* and *phyb-9* mutant, the correspondence between the PIF4 proteins and transcript becomes nonlinear due to negative feedback. Although we may expect lower transcripts due to higher PIF4 stability, a mild increase in PIF4 may not be enough to cross the threshold needed to induce the autoinhibition and lower the transcripts significantly. As autoinhibition is a dynamic process, the transcript levels for *PIF4-OE1* and *phyb-9* mutant may become temporarily higher than or comparable to the WT, highlighting the complexity of such regulation. Nevertheless, *PIF4-OE2*, which has a much higher protein level than *PIF4-OE1* and Col-0 (Figure 1A), consistently show lower transcript accumulation and GUS activity irrespective of photoperiod (Figure 1).

8. Author can provide the western for CCA1/LHY1 or SHB1 at SD ZT23 especially in cop1 mutant background, which are the known positive regulators of PIF4 expression. Also, in cop1 mutants we would expect high abundance of HY5 in the nucleus. HY5 inhibits the targets of PIF4. Because of these factors won't we expect promoter activity in cop1 mutant even if PIF4 is not stable. No visible GUS stain in cop1 background compared to WT and pif4-101 needs further experimental evidence.

Author response: Thank you for the suggestion, and we agree with the reviewer's hypothesis on this point. As suggested, we performed gene expression analysis for *CCA1*, *LHY* and *SHB1* and western blot for CCA1. Our RT-qPCR data revealed that the

transcript levels of *CCA1* and *LHY* were significantly elevated in the *cop1-4* and *cop1-6* mutants compared to wild-type (WT) (Figure S4M and S4N). Interestingly, the transcript levels of *CCA1* and *LHY* are also higher in the *35S:COP1* line than in WT (Figure S4M and S4N). Similarly, the *SHB1* transcript level in the *cop1-6* mutant and *35S:COP1* was elevated, but in the *cop1-4 mutant*, it was comparable to WT (Figure S4O). Moreover, immunoblot data also suggest that the CCA1 protein accumulation was higher in the *cop1-4*, *cop1-6* mutants and less in the *35S:COP1* compared to the WT (Figure S4P). These results suggest that the reduced *PIF4* promoter activity in the *cop1* mutants is probably not due to reduced *CCA1/LHY/SHB1* transcript levels or reduced *CCA1* protein levels.

Furthermore, we performed western blot analysis to check the HY5 protein levels under SD photoperiod. We observed elevated protein accumulation in *cop1-4* and *cop1-6* mutants compared to WT (Figure S4Q). This observation is in line with previous reports that COP1 acts in the nucleus to repress light-responsive gene expression and developmental processes [17] and inhibits HY5 activity through protein degradation [18, 19]. Moreover, there has been no report suggesting that HY5 can act upstream of PIF4, directly influencing its transcription. Nevertheless, HY5 has been shown to inhibit PIF4 targets [20]. Thus, HY5 likely has less influence on directly inhibiting *PIF4* gene expression, but it inhibits the expression of PIF4 targets. Collectively, these results suggest that reduced *PIF4* promoter activity in the *cop1* mutants is likely independent of CCA1/LHY, SHB1, and HY5. Our study reveals that COP1 in general positively influences the *PIF4* gene expression and *pPIF4:GUS* promoter activity (Figure 3). However, the exact mechanism of how COP1 influences *PIF4* protomer activity is still largely unknown, and this will be investigated in the future.

9. Fig 3A and 3B: Authors have observed that PIF4 promoter activity as well as PIF4 transcript levels are significantly reduced in 35S:COP1 than in WT, due to PIF4 concentration dependent autoinhibition. Since COP1 positively regulate PIF4 promoter activity, there should be a threshold PIF4 concentration below which COP1 positive regulation dominates more than the PIF4 mediated autoinhibition resulting in an increased pPIF4-GUS activity. In this regard, was the GUS activity measured in seedlings younger than 6 days old?

Author response: Thank you for the insightful comments. Following the reviewer's suggestion, we checked the GUS staining and quantitative measurement of *PIF4* promoter activity in three to six-day-old seedlings in the COP1 overexpression line (*35S:COP1*) (Figure S4E and S4F). Interestingly, the *PIF4* promoter activity in *35S:COP1* was comparable to that of the wild type on day 3, and then the promoter activity in *35S:COP1* became systematically reduced compared to wild-type (Figure S4F). Thus, our data suggest that though the *PIF4* promoter activity in *35S:COP1* was similar to wild-type at early stages, subsequently, the PIF4 level quickly goes above the threshold due to elevated COP1 level in *35S:COP1*, likely leading to stronger PIF4 autoinhibition at later stages.

10. The justification behind including rosette biomass and seed yield in the manuscript without enough experimental support is not clear.

Author response: We wanted to check the influence of PIF4-dependent autoinhibition on the downstream growth-promoting genes and subsequently on rosette biomass and seed yield. We have shown that PIF4 target genes that promote growth (such as *YUC8*

and *IAA29*) are significantly downregulated in the PIF4 overexpression lines (*PIF4-OE1* and *PIF4-OE2*) at 27°C than at 22°C, although higher temperature promotes their expression in the wild-type (Figure 7A,B). This opposing trend can be explained by the PIF4-mediated autoinhibition. We observed the same trend in another growth-promoting gene *XTR7* (Figure S6L). Since the autoinhibition is stronger in *PIF4-OE1* and *PIF4-OE2* than in the wild-type, the PIF4 target genes are, in turn, less expressed in the overexpression lines to optimize growth. Also, we observed less biomass and seed yield in *PIF4-OE1* and *PIF4-OE2* compared to wild-type in both 22°C and 27°C.

Minor Comments:

1. In introduction last paragraph, many sentences are redundant, kindly reframe the paragraph.

Author response: Thank you for your suggestion. We have rephrased the introduction in our revised manuscript.

2. In Figure 5I, kindly check the statistics of 35S:PHYB in 22'C and 27'C, they do not seem to be in the same statistical group.

Author response: We apologize for the typo. We have corrected this in our revised manuscript.

3. In Figure 6 A, WT 22 and WT 28, protein size of PIF4 band is looking different. Kindly check carefully.

Author response: Thank you for pointing this out. We have corrected this.

4. In all the blots pif4 is not showing the non-specific bands seen in Col-0, why?

Author response: The PIF4-specific bands come around 60 kDa, where we could not detect bands for the *pif4* mutant in the blots. The messy-looking bands for PIF4 are likely due to both phosphorylated and non-phosphorylated forms of PIF4, which could result in multiple bands corresponding to different modified forms of the protein, and we could not see those bands in the mutant. We have repeated our western blots at least three times and observed similar results. A similar band pattern for PIF4 was also observed in an earlier report [7, 8].

5. In Fig S1O ZT23 is chosen for SD and in Figure S1M, ZT4 was chosen for SD, kindly explain.

Author response: We chose the above ZT time points since PIF4 peaks its expression at ZT23 in SD and at ZT4 in LD. As already known in the literature [14, 21-23], the PIF4 activity generally increases in the SD than in the LD. Thus, according to our hypothesis of PIF4 autoinhibition, we would expect higher inhibition strength in SD than in LD. Comparisons between LD and SD further suggest that PIF4 autoinhibition is regulated in a photoperiod-dependent manner (Figures S1O and S1P). Consistent with this, we found lower GUS activity when we compared SD (ZT23) and LD (ZT4) in the WT and *pif4* mutant (Figures S1O). Moreover, at ZT4, we found increased activity in LD than in SD in both WT and *pif4* mutants (Figure S1P).

6. What is the COP1 expression level in cop1 mutant and overexpressor crossed with pPIF4:GUS?

Author response: Thank you for the suggestion. We have checked the COP1 protein and transcript levels in the *cop1* mutants (*cop1-4* and *cop1-6)* and *35S:COP1* crossed with *pPIF4:GUS* (Figure S4C,D). Our data suggested that the *COP1* mRNA level in *cop1-6* was similar to WT as reported before (Nieto et al., 2020, Fig. 2b), whereas the *COP1* expression was slightly reduced in the *cop1-4* mutant, and significantly increased in *35S:COP1* compared to WT (Figure S4C). Further, the immunoblot analysis by using native COP1 antibody showed more protein accumulation in *35S:COP1* compared to WT (Figure S4D). Moreover, in both *cop1-4* and *cop1-6,* COP1 protein levels were markedly reduced (Figure S4D). Thus, our immunoblot data suggest that reduced COP1 activity in the mutants results in reduced *PIF4* promoter activity, as expected (Figure 3A,B). However, elevated COP1 activity in the *35S:COP1* also results in reduced promoter activity, likely due to higher PIF4 protein accumulation and corresponding feedback inhibition (Figure 3B,E).

7. Figure 6 legend: "All the immunoblotting experiment was performed at teh same time and maintained the identical conditions thrpughout the procedure." Kindly check and correct the spelling.

Author response: We apologize for the typo. Now we have corrected in our revised manuscript.

8. Figure 7A and B: How many days old seedlings were harvested in order to check the relative expression levels of the temperature induced and growth responsive genes YUC8 and IAA29 ?

Author response: We have used six-day-old seedlings to check the gene expression, and now mentioned in the figure legend in our revised manuscript.

9. Why was PIF4 particularly chosen for the study? Were any other PIFs as well checked for their role in autoregulation similarly to PIF4?

Author response: Even though most PIF family members play a key role in controlling hypocotyl growth, PIF4 is the major regulator of growth that integrates both light and temperature and endogenous cues [24-26]. This is the main reason for using PIF4 as a model in investigating whether this important regulator undergoes any autoregulation. Nevertheless, we agree with the reviewer that it will be interesting to investigate other key PIF proteins, such as PIF7 and PIF3, in potential autoregulatory mechanisms.

References

1. Qiu Y, Pasoreck EK, Yoo CY, He J, Wang H, Bajracharya A, et al. RCB initiates Arabidopsis thermomorphogenesis by stabilizing the thermoregulator PIF4 in the daytime. Nature Communications. 2021;12(1):2042. doi: 10.1038/s41467-021-22313-x.
2. Tan W, Chen J, Yue X, Chai S, Liu W, Li C, et al. The heat response regulators HSFA1s promote <i>Arabidopsis</i> thermomorphogenesis via stabilizing PIF4 during the day. Sci Adv. 2023;9(44):eadh1738. doi: doi:10.1126/sciadv.adh1738.

3. Andrási N, Pettkó-Szandtner A, Szabados L. Diversity of plant heat shock factors: regulation, interactions, and functions. J Exp Bot. 2021;72(5):1558-75. doi: 10.1093/jxb/eraa576. PubMed PMID: 33277993.
4. Han X, Yu H, Yuan R, Yang Y, An F, Qin G. Arabidopsis Transcription Factor TCP5 Controls Plant Thermomorphogenesis by Positively Regulating PIF4 Activity. iScience. 2019;15:611-22. Epub 20190508. doi: 10.1016/j.isci.2019.04.005. PubMed PMID: 31078552; PubMed Central PMCID: PMCPMC6548983.
5. Sun Q, Wang S, Xu G, Kang X, Zhang M, Ni M. SHB1 and CCA1 interaction desensitizes light responses and enhances thermomorphogenesis. Nat Commun. 2019;10(1):3110. Epub 20190715. doi: 10.1038/s41467-019-11071-6. PubMed PMID: 31308379; PubMed Central PMCID: PMCPMC6629618.
6. Ding L, Wang S, Song ZT, Jiang Y, Han JJ, Lu SJ, et al. Two B-Box Domain Proteins, BBX18 and BBX23, Interact with ELF3 and Regulate Thermomorphogenesis in Arabidopsis. Cell Rep. 2018;25(7):1718-28 e4. doi: 10.1016/j.celrep.2018.10.060. PubMed PMID: 30428343.
7. Fang W, Vellutini E, Perrella G, Kaiserli E. TANDEM ZINC-FINGER/PLUS3 regulates phytochrome B abundance and signaling to fine-tune hypocotyl growth. Plant Cell. 2022;34(11):4213-31. doi: 10.1093/plcell/koac236. PubMed PMID: 35929801; PubMed Central PMCID: PMCPMC9614508.
8. Dong X, Yan Y, Jiang B, Shi Y, Jia Y, Cheng J, et al. The cold response regulator CBF1 promotes <i>Arabidopsis</i> hypocotyl growth at ambient temperatures. The EMBO Journal. 2020;39(13):e103630. doi: https://doi.org/10.15252/embj.2019103630.
9. Zhai H, Xiong L, Li H, Lyu X, Yang G, Zhao T, et al. Cryptochrome 1 Inhibits Shoot Branching by Repressing the Self-Activated Transciption Loop of PIF4 in Arabidopsis. Plant Commun. 2020;1(3):100042. Epub 20200326. doi: 10.1016/j.xplc.2020.100042. PubMed PMID: 33367238; PubMed Central PMCID: PMCPMC7748022.
10. Nieto C, Catalan P, Luengo LM, Legris M, Lopez-Salmeron V, Daviere JM, et al. COP1 dynamics integrate conflicting seasonal light and thermal cues in the control of Arabidopsis elongation. Sci Adv. 2022;8(33):eabp8412. Epub 20220819. doi: 10.1126/sciadv.abp8412. PubMed PMID: 35984876; PubMed Central PMCID: PMCPMC9390991.
11. Gangappa SN, Berriri S, Kumar SV. PIF4 Coordinates Thermosensory Growth and Immunity in Arabidopsis. Curr Biol. 2017;27(2):243-9. Epub 20161229. doi: 10.1016/j.cub.2016.11.012. PubMed PMID: 28041792; PubMed Central PMCID: PMCPMC5266789.
12. Nozue K, Covington MF, Duek PD, Lorrain S, Fankhauser C, Harmer SL, et al. Rhythmic growth explained by coincidence between internal and external cues. Nature. 2007;448(7151):358-U11. doi: 10.1038/nature05946. PubMed PMID: WOS:000248118300059.
13. Nomoto Y, Kubozono S, Yamashino T, Nakamichi N, Mizuno T. Circadian clock- and PIF4-controlled plant growth: a coincidence mechanism directly integrates a hormone signaling network into the photoperiodic control of plant architectures in Arabidopsis thaliana. Plant Cell Physiol. 2012;53(11):1950-64. Epub 20121004. doi: 10.1093/pcp/pcs137. PubMed PMID: 23037003.
14. Nomoto Y, Kubozono S, Miyachi M, Yamashino T, Nakamichi N, Mizuno T. A circadian clock- and PIF4-mediated double coincidence mechanism is implicated in the thermosensitive photoperiodic control of plant architectures in Arabidopsis thaliana. Plant Cell Physiol. 2012;53(11):1965-73. Epub 20121004. doi: 10.1093/pcp/pcs141. PubMed PMID: 23037004.
15. Soy J, Leivar P, González-Schain N, Sentandreu M, Prat S, Quail PH, et al. Phytochrome-imposed oscillations in PIF3 protein abundance regulate hypocotyl growth under diurnal

light/dark conditions in Arabidopsis. Plant J. 2012;71(3):390-401. Epub 20120611. doi: 10.1111/j.1365-313X.2012.04992.x. PubMed PMID: 22409654; PubMed Central PMCID: PMCPMC3465574.

16. Yamashino T, Nomoto Y, Lorrain S, Miyachi M, Ito S, Nakamichi N, et al. Verification at the protein level of the PIF4-mediated external coincidence model for the temperature-adaptive photoperiodic control of plant growth in Arabidopsis thaliana. Plant Signal Behav. 2013;8(3):e23390. Epub 20130108. doi: 10.4161/psb.23390. PubMed PMID: 23299336; PubMed Central PMCID: PMCPMC3676505.
17. von Arnim AG, Deng XW. Light inactivation of Arabidopsis photomorphogenic repressor COP1 involves a cell-specific regulation of its nucleocytoplasmic partitioning. Cell. 1994;79(6):1035-45. doi: 10.1016/0092-8674(94)90034-5. PubMed PMID: 8001131.
18. Ang LH, Deng XW. Regulatory hierarchy of photomorphogenic loci: allele-specific and light-dependent interaction between the HY5 and COP1 loci. Plant Cell. 1994;6(5):613-28. doi: 10.1105/tpc.6.5.613. PubMed PMID: 8038602; PubMed Central PMCID: PMCPMC160463.
19. Osterlund MT, Hardtke CS, Wei N, Deng XW. Targeted destabilization of HY5 during light-regulated development of Arabidopsis. Nature. 2000;405(6785):462-6. doi: 10.1038/35013076. PubMed PMID: 10839542.
20. Gangappa SN, Kumar SV. DET1 and HY5 Control PIF4-Mediated Thermosensory Elongation Growth through Distinct Mechanisms. Cell Rep. 2017;18(2):344-51. doi: 10.1016/j.celrep.2016.12.046.
21. Huang H, Yoo CY, Bindbeutel R, Goldsworthy J, Tielking A, Alvarez S, et al. PCH1 integrates circadian and light-signaling pathways to control photoperiod-responsive growth in Arabidopsis. Elife. 2016;5:e13292. doi: 10.7554/eLife.13292.
22. Li N, Zhang Y, He Y, Wang Y, Wang L. Pseudo Response Regulators Regulate Photoperiodic Hypocotyl Growth by Repressing PIF4/5 Transcription1 [OPEN]. Plant Physiology. 2020;183(2):686-99. doi: 10.1104/pp.19.01599.
23. Nozue K, Covington MF, Duek PD, Lorrain S, Fankhauser C, Harmer SL, et al. Rhythmic growth explained by coincidence between internal and external cues. Nature. 2007;448(7151):358-61. doi: 10.1038/nature05946.
24. Leivar P, Quail PH. PIFs: pivotal components in a cellular signaling hub. Trends Plant Sci. 2011;16(1):19-28. Epub 2010/09/14. doi: 10.1016/j.tplants.2010.08.003. PubMed PMID: 20833098; PubMed Central PMCID: PMCPMC3019249.
25. Casal JJ, Balasubramanian S. Thermomorphogenesis. Annu Rev Plant Biol. 2019;70:321-46. Epub 2019/02/21. doi: 10.1146/annurev-arplant-050718-095919. PubMed PMID: 30786235.
26. Lee S, Zhu L, Huq E. An autoregulatory negative feedback loop controls thermomorphogenesis in Arabidopsis. PLoS Genet. 2021;17(6):e1009595. Epub 20210601. doi: 10.1371/journal.pgen.1009595. PubMed PMID: 34061850; PubMed Central PMCID: PMCPMC8195427.
